# Development and validation of ultra-performance liquid chromatographic method with tandem mass spectrometry for determination of lenalidomide in rabbit and human plasma

**DOI:** 10.1186/1752-153X-7-7

**Published:** 2013-01-14

**Authors:** Muzaffar Iqbal, Tanveer A Wani, Nasr Y Khalil, Ibrahim A Darwish

**Affiliations:** 1 Department of Pharmaceutical Chemistry, College of Pharmacy, King Saud University, P.O. Box 2457, 11451, Riyadh, Saudi Arabia

**Keywords:** Lenalidomide, Ultra performance liquid chromatography, Tandem mass spectrometry, Pharmacokinetic and toxicokinetic, High throughput analysis

## Abstract

**Background:**

Lenalidomide (LND) is a potent novel thalidomide analog which demonstrated remarkable clinical activity in treatment of multiple myeloma disease via a multiple-pathways mechanism. Validated sensitive method with high throughput is required for the determination of lenalidomide for pharmacokinetics and toxicokinetic studies. Ultra performance liquid chromatography-tandem mass spectrometry (UPLC-MS/MS) is a preeminent analytical tool for rapid biomedical analysis.

**Results:**

A simple, highly sensitive UPLC-MS/MS method was developed and validated for the determination of LND in rabbit and human plasma. After a simple protein precipitation using methanol, LND and carbamazepine (IS) were separated on Acquity UPLC BEH™ C_18_ column (50 × 2.1 mm, i.d. 1.7 μm, Waters, USA) using a mobile phase consisted of acetonitrile:water:formic acid (65:35:0.1%, v/v/v) pumped at a flow rate of 0.2 mL/min. LND and IS were eluted at 0.71 and 1.92 min, respectively. The mass spectrometric determination was carried out using an electrospray interface operated in the positive mode with multiple reaction monitoring (MRM) mode. The precursor to product ion transitions of *m*/*z* 260.1 > 149.0 and *m*/*z* 237.0 > 179.0 were used to quantify LND and IS, respectively. The method was linear in the concentration range of 0.23–1000 ng/mL with a limit of quantitation of 0.23 ng/mL. All the validation parameters were in the ranges acceptable by the guidelines of analytical method validation.

**Conclusion:**

The proposed UPLC-MS/MS method is simple, rapid and highly sensitive, and hence it could be reliable for pharmacokinetic and toxicokinetic study in both animals and humans.

## Background

Lenalidomide (LND) is an oral immunomodulatory drug with antiangiogenic and antineoplastic properties. It structurally resembles thalidomide but has an improved toxicity profile and more potent immunomodulatory activity [1,2]. LND demonstrated remarkable clinical activity in treatment of multiple myeloma disease [3-7] via a multiple-pathways mechanism [8-11]. LND is rapidly absorbed with the maximal plasma concentration occurring at a median time of 0.6 to 1.5 hours after oral administration in healthy human subjects. Co-administration of LND with food delays its absorption, but it does not alter the extent of absorption. LND displays linear pharmacokinetics in healthy subjects as well as in patients with normal renal function. The plasma exposure of LND is dose proportional and it does not accumulate with multiple doses. Approximately two thirds of orally administered LND is eliminated as unchanged in urine, likely via glomerular filtration and active tubular secretion. The mean terminal half-life (t_1/2_) is 3 to 4 hours [12,13].

Many analytical methods have been reported for the determination of LND. Two spectrophotometric [14] and one fluorimetric [15] methods were developed for the determination LND in bulk material and in capsules. Besides, two HPLC methods have been developed for analysis of bulk material of LND and its related impurities [16] and in capsules [17]. Two analytical reports were also reported describing the determination of the LND in biological samples (plasma). The first one employed liquid chromatography-mass spectrometry using single ion monitoring with a limit of quantitation of 5 ng/mL[18]. The second method included the simultaneous determination of LND and flavopiridol by liquid chromatography-mass spectrometry (LCMS/MS) using a single reaction monitoring (SRM) and a gradient elution [19]. Both methods have the disadvantages of long run time of 8 and 10 min respectively which does not meet the requirement of high throughput and speedy analysis of biosamples in clinical laboratories. Therefore, an alternative validated method with both high sensitivity and high throughput will be an advance for greater efficiency in pre-clinical pharmacokinetics, toxicokinetic, and clinical studies.

Among the currently available bio-analytical techniques, ultra-performance liquid chromatography (UPLC) has gained a considerable attention in recent years and has been emerged as the preeminent analytical tool for pharmaceutical and biomedical analysis because of its high speed, low time consumption, better resolution and better sensitivity. The present study describes the development and validation of an UPLC method coupled with tandem mass spectrometry (UPLC-MS/MS) for the determination of LND in both rabbit and human plasma. The method was found to be simple, highly sensitive, has high-throughput and reproducible.

## Experimental

### Material and method

Lenalidomide, free base (3-(4-amino-1-oxo-1,3-dihydro-2*H*-isoindol-2-yl) piperidine-2,6-dione) (LC Laboratories®, Woburn, MA, USA ) was obtained and used as received; its purity was 100.2 ± 1.25%. Carmamazeipine (purity > 98%) was obtained from Tabuk Pharmaceutical Manufacturing Co. (Tabouk, Saudi Arabia). HPLC-grade acetonitrile and methanol were obtained from Winlab Laboratory, UK. Formic acid was obtained from BDH Laboratory, England. All other reagents were of analytical grade unless stated otherwise. All aqueous solutions were prepared using water that was purified using Milli-QR Gradient A10R (Millipore, Moscheim Cedex, France) having pore size 0.22 μm.

### Apparatus and operating condition

#### Liquid chromatography

The chromatography was performed on an ACQUITY ™ UPLC system (Waters Corp., Milford, MA, USA). The UPLC system included quaternary solvent manager, a binary pump, degasser, autosampler with an injection loop of 10 μL and a column heater-cooler. The separation was performed on Acquity UPLC BEH™ C_18_ column (50 × 2.1 mm, i.d., 1.7 μm, Waters, USA) maintained at 45°C. The mobile phase was composed of acetonitrile:water:formic acid (65:35:0.1%, v/v/v) at a flow rate of 0.2 mL/min. The injection volume was 5 μL in partial loop mode and the temperature of the autosampler was kept at 4°C.

#### Mass spectrometric conditions

A triple-quadrupole tandem mass spectrometer (Micromass® Quattro micro™ Waters Corp., Milford, MA, USA) equipped with electrospray ionization (ESI) interface was used for analytical detection. The ESI source was operated in positive ionization mode. Quantification was performed using multiple reaction monitoring (MRM) of the transitions of m/z 260.1 > 149.0 for LND and m/z 237.0 > 179.0 for carbamazepine, with the dwell time of 0.16 s. Nitrogen was used as a desolvating gas at a flow rate of 600 L/h. The desolvating temperature was 350°C whereas source temperature was 150°C. The collision gas (argon) flow was 0.1 mL/min and capillary voltage was set at 4 kV. The MS analyzer parameters were as follows: LM1 and HM1 resolution 14.0 and 14 respectively; ion energy 1; LM2 and HM2 resolution 10.0 and 14.0 respectively, ion energy 2. The compound parameters like cone voltage and collision energy were optimized and set at 27 V and 19 eV for LND and 36 V and 36 eV for IS respectively. The Mass Lynx software (Version 4.1, SCN 714) was used to control the UPLC-MS/MS system and data was collected and processed using TargetLynx™ program.

### Calibration standards and quality control samples

A standard stock solution of LND and carbamazepine internal standard (IS) were prepared by dissolving the compound in dimethylsulphoxide and methanol, respectively to give a final concentrations of 1 mg/mL. The solutions were kept in the refrigerator and could be used for 15 days from the date of preparation. Stock solution of LND was used for calibration standards and quality control (QC) samples, respectively. Working solutions of LND were prepared in methanol:water (50:50, v/v) to obtain aqueous calibration curve standards of 11.52-50000 ng/mL. A 20 μL aliquot of each working solution was added to blank human/rabbit plasma to yield calibration standards ranging from 0.23 to 1000 ng/mL. QC samples at four different concentrations (0.26, 1.28, 64 and 800 ng/mL) were prepared in a similar manner as the calibration standards and they are treated as LLOQ QC, LQC, MQC and HQC respectively. Spiked plasma calibration standards and quality control samples were kept at −80°C until assayed or used for validating the assay procedures. The IS working solution (5 μg/mL) for routine use was prepared by diluting the carbamazepine stock solution in methanol:water (50:50, v/v) and kept at room temperature (25 ± 2°C).

### Sample preparation

Plasma samples stored at around −80°C were thawed, left for 1 hour and vortex for 30 sec on room temperature before extraction to ensure homogeneity. To 200 μL of plasma sample 20 μL of working standard and 25 μL (5 μg/mL) of IS (except blank sample) was added. The samples were vortex mixed for about 30 sec and 750 μL of methanol was added to it. The samples were again vortex mixed gently for 1.5 min and then cold centrifuged for 10 min at 10000 rpm. After centrifugation, 400 μl of supernatant was transferred into HPLC vial, and 5 μL volumes (in partial loop with needle over fill mode) of the sample were subjected to the analysis by UPLC–MS/MS.

### Method validation

A full method validation was performed according to guidelines set by the United States Food and Drug Administration (US-FDA) and European Medicines Agency (EMEA) guidelines [20,21]. The validation of this procedure was performed in rabbit plasma in order to evaluate the method in terms of selectivity, linearity of response, accuracy, precision, recovery, dilution integrity and stability of analytes during both short-term sample processing and long-term storage. Selectivity, linearity, accuracy and precision exercise was also performed in human plasma.

#### Selectivity and specificity

The selectivity of the method towards endogenous plasma matrix components, metabolites and component medications was assessed in rabbit and human blank plasma. Among the analyzed batch, plasma batch showing no or minimal interference at the retention time of analyte and internal standard was selected. They were processed and analyzed using the proposed extraction protocol spiked with standard LND at LLOQ level (0.23 ng/mL) and carbamazepine (IS) at 100 ng/mL level.

#### Linearity and standard curve

The linearity of the method was determined by analysis of standard plots associated with an eight point standard calibration curve (0.23-1000 ng/mL). Calibration curves from accepted three precision and accuracy batches were used to establish linearity. Curves were best fitted using a least square linear regression model y = mx + b, weighted by 1/x^2^, in which y is the peak area ratio, m is slope of the calibration curve, b is the y-axis intercept of the calibration curve and x is the analyte (LND) concentration. Back-calculations were made from these curves to determine the concentration of LND in each calibration standards and the resulting calculated parameters were used to determine concentrations of analyte in quality control samples. The determination coefficient r^2^ > 0.98 was desirable for all the calibration curves. The lowest standard on the calibration curve was to be accepted as the lower limit of quantification (LLOQ), if the analyte response was at least five times more than that of drug free (blank) extracted plasma. In addition, the analyte peak of LLOQ sample should be identifiable, discrete, and reproducible with accuracy within ± 20%and a precision ≤ 20%. The deviation of standards other than LLOQ from the nominal concentration should not be more than ± 15.0%.

#### Precision and accuracy

Intra- and inter-day accuracies expressed as a percentage of deviation from the respective nominal value. The precision of the assay was measured by the percent coefficient of variation (%CV) at four concentrations in both rabbit and human plasma. Intra-day precision and accuracy were assessed by analyzing six replicates of the quality control samples at four levels (quality control) during a single analytical run. The inter-day precision and accuracy were assessed by analyzing 18 replicates of the quality control samples at each level through three precision and accuracy batches runs on 2 consecutive validation days. The deviation at each concentration level from the nominal concentration was expected to be within ±15.0%except LLOQ QC, for which it should not be more than 20.0%. Similarly, the mean accuracy should not deviate by ± 15.0%except for the LLOQ QC where it can be ± 20.0%of the nominal concentration.

#### Extraction recovery and matrix effect

Standard aqueous quality control stock of LND each at low, medium, and high concentration levels were spiked in plasma and millipore water separately, the later being considered as unextracted QC samples. Six replicates of each QC plasma samples were processed as usual and analyzed along with six replicates of unextracted standard QC samples by applying correction factor to nullify dilution of extracted samples during plasma processing. Six replicates of aqueous carbamazepine were also run for the recovery of carbamazepine.

#### Stability and dilution integrity evaluation

Stability of LND in plasma was assessed by analyzing six replicates of QC samples at low and high concentrations under a variety of storage and processing conditions. Six aliquots of each low and high concentration quality control samples were taken to evaluate the bench top stability (short term stability), freeze thaw stability, autosampler storage stability and long term stability. Bench-top stability was assessed after exposure of the plasma samples to room temperature for ∼ 6 h, which exceeds the residence time of the sample processing procedures. The freeze–thaw stability was evaluated after undergoing three freeze (at around −80°C)–thaw (room temperature) cycles. The autosampler storage stability was determined by storing the reconstituted QC samples for ∼ 48 h under autosampler condition (maintained at 8°C) before being analyzed. Long-term stability was assessed after storage of the test samples at around −80°C for 30 days. The working solutions and stock solutions of LND and the IS were also evaluated for stability at room temperature for 12 h and at refrigerator temperature (below 10°C) for 15 days. All stability exercises were performed against freshly spiked calibration standards. The samples were considered stable in plasma at each concentration if the deviation from the mean calculated concentration of stability quality control samples was within ±15%.

Dilution integrity exercise was carried out to ensure the integrity of analyte in those samples which are beyond upper limit of the standard curve and need to be diluted. A fresh stock of LND was prepared and spiked in plasma to get a conc. level of 1.8 times of highest standard of the usual calibration standard. It was then diluted 2 times and 4 times with the same plasma. Six aliquots of both dilutions were processed along with freshly spiked calibration standards and analyzed by back calculation using regression equation obtained. The integrity of the samples were considered to be maintained if -%nominal is within ± 15%of nominal values and %CVs ≤ 15%at both diluted levels.

## Result and discussion

### Optimization of chromatographic condition

**Figure 1:**
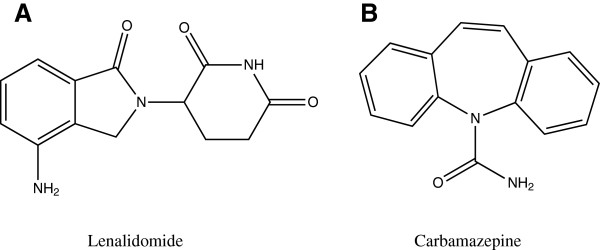
Chemical structure of lenalidomide [A] and carbamazepine (IS) [B].

Initial feasibility experiments of various mixture(s) of organic solvents such as acetonitrile and methanol along with pure water; both having 0.1%formic acid along with altered flow-rates (in the range of 0.20–0.4 mL/min) was performed to optimize an effective chromatographic resolution of LND and IS; their chemical structures are given in Figure 1. The best resolution of peaks were achieved with an isocratic elution by a mobile phase comprising acetonitrile:water:formic acid (65:35:0.1%, v/v/v) at a flow-rate of 0.2 mL/min, on Acquity UPLC BEH™ C18 column (50 × 2.1 mm, i.d. 1.7 μm). The selected conditions were found to be suitable for the determination of electrospray response for LND and IS.

**Figure 2:**
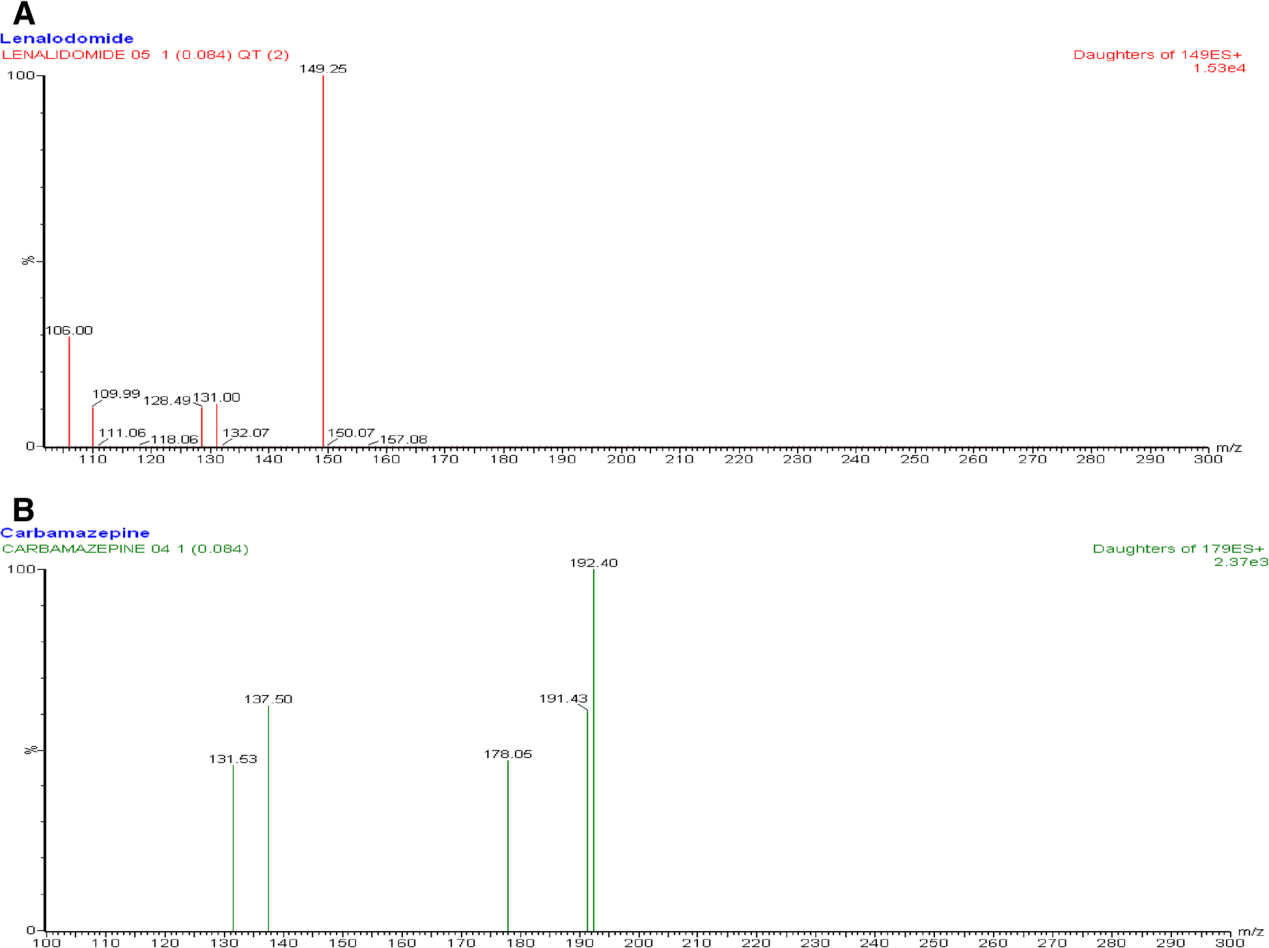
The product ion spectra of lenalidomide [A] and carbamazepine (IS) [B].

UPLC-MS/MS operation parameters were carefully optimized for the determination of LND. Both analyte and IS were detected by tandem mass spectrometry using MRM of precursor–product ion transitions with 0.160 s dwell time, at m/z 260.1 > 149.0 for LND and m/z 237.0 > 179.0 for carbamazepine. A standard solution (100 ng/mL) of LND and the carbamazepine were directly infused along with the mobile phase into the mass spectrometer with ESI as the ionization source. The mass spectrometer was tuned initially in both positive and negative ionization modes for LND. It was observed that the signal intensity of positive ion was much higher than that of negative ion. Parameters, such as capillary and cone voltage, desolvation temperature, ESI source temperature and flow rate of desolvation gas and cone gas, were optimized to obtain the optimum intensity of deprotonated molecules of LND and IS for quantification. Among the parameters, capillary and cone voltage, especially cone voltage, were important parameters. The precursor ion intensities increased significantly when cone voltage was raised gradually. Lastly, analytes produced the strongest ion signals when cone voltage was set up at 27 V. While cone voltage exceeded 27 V, the ion signals decreased rapidly. The collision energy was investigated from 10 to 50 eV to optimize the response of product ion, and the best values were found to be 19 eV for the chosen product ions (m/z 149). For IS, although the most abundant fragment ion was observed at m/z 191.0, but m/z spectra at 179.0 at the optimum collision energy 36 eV give more resolved peak hence chosen for quantification. The product ion spectra of LND and IS were shown in Figure 2.

### Optimization of sample processing

Protein precipitation was used for sample preparation in this study. Protein precipitation can be helpful in producing a clean sample and avoiding endogenous substances in plasma with the analytes and IS onto the column and MS system. Clean samples are essential for minimizing ion suppression and matrix effect in UPLC–MS/MS analysis. Two organic solvents: methanol and acetonitrile in presence or absence of formic acid were evaluated. Finally methanol was found to be optimal, which can produce a clean chromatogram for a blank plasma sample and yield the highest recovery for the analytes from the rabbit plasma.

### Selectivity

**Figure 3:**
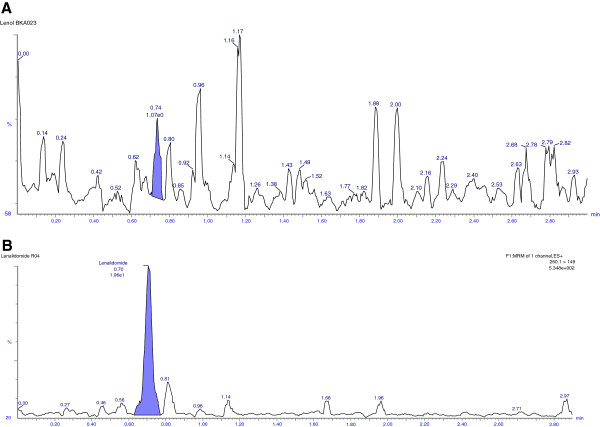
Representative chromatograms of blank [A] and LLOQ [B] of LND in rabbit plasma.

Selectivity of the method was assessed by comparing the chromatograms of both rabbit and human blank plasma with the corresponding spiked LLOQ sample. Only those lots which are under the acceptance criteria (< 20%in comparison to the spiked LLOQ and < 5%in comparison to IS area) were selected. This infers that there were no potential significant endogenous substances in plasma that interfered with the peaks of analyte and IS. Thus the method looks to be selective enough for determination of LND and carbamazepine in plasma. Representative chromatograms obtained from blank plasma showing no interference at the retention time of analyte and IS are shown in Figures 3 [A] and 4 [A], respectively. Representative chromatogram of LLOQ and IS are shown in Figures 3 [B] and 4 [B], respectively whereas representative chromatogram of LQC and HQC are shown in Figure 5 [A] and [B] respectively.

**Figure 4:**
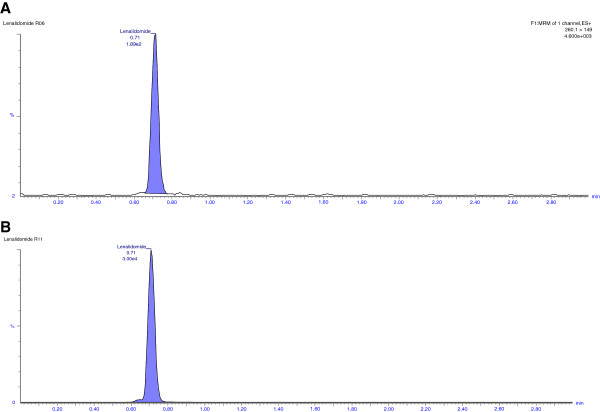
Representative chromatograms of carbamazepine (IS) in blank [A] and HQC [B] in rabbit plasma.

**Figure 5:**
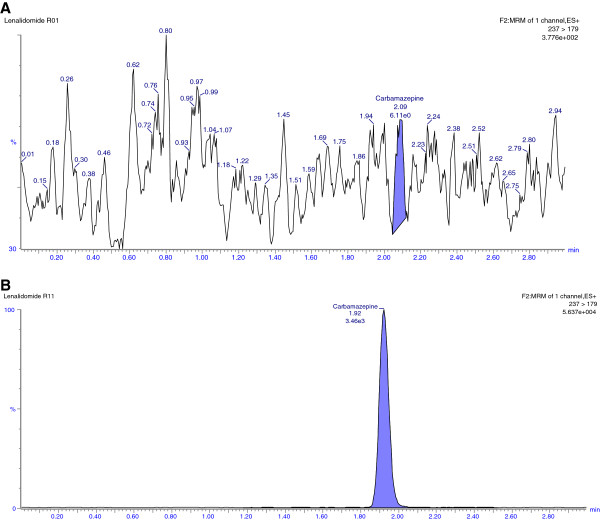
Representative chromatograms of LQC [A] and HQC [B] of LND in rabbit plasma.

### Linearity and sensitivity

The linearity of the method was determined by a weighted least square regression analysis of standard plot associated with an eight-point standard curve. The calibration curves were generated by plotting area ratio (LND/IS) as a function of LND concentration. It was found to be linear from 0.23 to 1000 ng/mL for LND in both rabbit and human plasma. The determination coefficients (r^2^) were consistently greater than 0.995 during the course of validation. The lower limit of quantification for this assay was 0.23 ng/mL in plasma. Representative LLOQ is sensitive enough to investigate the pharmacokinetic behavior of LND in both rabbit and human.

### Precision and accuracy

Table 1 summarizes the inter- and intra-day precision and accuracy values for QC samples. The coefficient of variation values of both intra- and inter-day results of rabbit plasma were 2.14 - 12.2 and 4.62 - 9.67%, respectively, whereas the intra- and inter-day accuracies were 91.4–108 and 95.1 – 104%, respectively. Similarly coefficients of variation values of both intra- and inter-day results were 1.97 - 9.61 and 4.54-9.72%respectively whereas intra- and inter-day accuracies were 93.5–113%and 98.3 – 111%, respectively in human plasma. These results indicate that the method has good precision and accuracy and are within the acceptance limit of < 15%and ± < 15%for precision and accuracy respectively.

**Table 1:** Intra- and inter-day precision and accuracy of LND in rabbit and human plasma

**Nominal conc. (ng/mL)**	**Run**	**Rabbit plasma**			**Human plasma**		
		**Measured conc. (ng/mL ± SD)**	**Precision (CV,%)**	**Accuracy (recovery,%)**	**Measured conc. (ng/mL ± SD)**	**Precision (CV,%)**	**Accuracy (recovery,%)**
	*Intraday variation (six replicate at each concentration)*						
0.26	1	0.27 ± 0.02	6.98	103	0.29 ± 0.01	3.60	110
	2	0.26 ± 0.03	9.51	101	0.29 ± 0.03	9.61	109
	3	0.28 ± 0.03	12.2	108	0.30 ± 0.03	9.53	113
1.28	1	1.30 ± 0.07	6.74	101	1.32 ± 0.08	5.80	103
	2	1.32 ± 0.04	3.09	103	1.34 ± 0.05	3.51	105
	3	1.36 ± 0.06	4.78	106	1.37 ± 0.06	4.16	107
64.0	1	59.3 ± 5.35	9.02	92.7	59.8 ± 5.95	9.94	93.5
	2	64.8 ± 4.26	6.57	101	64.8 ± 5.42	8.36	101
	3	58.5 ± 6.19	10.60	91.4	63.8 ± 5.91	9.26	99.7
800	1	832 ± 17.70	2.14	104	814 ± 43.60	5.36	102
	2	778 ± 19.30	2.48	97.2	790 ± 34.50	4.37	98.8
	3	775.8 ± 38.60	4.97	97.0	769 ± 15.10	1.97	96.1
	*Intraday variation (18 replicates at each concentration)*						
0.26		0.27 ± 0.03	9.67	104	0.29 ± 0.03	9.72	111
1.28		1.32 ± 0.06	4.80	103	1.34 ± 0.06	4.54	105
64		60.9 ±5.80	9.49	95.1	62.9 ± 5.91	9.40	98.3
800		795 ±36.80	4.62	99.4	791 ± 36.60	4.62	99.2

### Recovery

Table 2 summarizes the percentage recovery of LND and carbamazepine (IS). The percentage recoveries (mean ± SD) of LND obtained from plasma at three QC concentration levels (1.28, 64 and 800 ng/mL), were 71.4 ± 7.9, 70.5 ± 6.4 and 72.2 ± 8.0%, respectively. The mean recovery for the carbamazepine (IS) at the concentration employed was 75.5 ±7.6%. This result indicates that the extraction efficiency for LND using protein precipitation method was satisfactory, consistent and concentration independent.

### Stability and dilution integrity

The stabilities of LND were investigated at two concentrations of QC samples (low and high concentrations) to cover expected conditions during analysis, storage and processing of all samples, which include the stability data from various stability exercises like in-injector, bench-top, freeze/thaw and long-term stability tests. The stability results summarized in Table 3 indicate that LND spiked into rabbit plasma was stable for at least 6.0 h at room temperature, for at least 48 h in final extract at 8°C under autosampler storage condition, for 30 days at around −80°C, and during three freeze–thaw cycles when stored at around −80°C and thawed to room temperature. These results indicate that there is no significant degradation of LND under the storage or handling conditions evaluated. The stock solutions and working standard of LND and IS were also stable for 15 days at refrigerator temperature (below 10°C) and for 12 h at room temperature. Therefore, LND was deemed to be stable upto 30 days at −80°C in spiked plasma and up to 15 days in aqueous solution in refrigerator, as reported previously [19].

**Table 2:** Recovery data of LND (three QC samples) and carbamazepine in rabbit plasma

**Compound**	**Nominal conc. (ng/mL)**	**Recovery (%± SD )**
LND (analyte)	1.28	71.4 ± 7.9
	64	70.5 ± 6.4
	800	72.5 ± 8.0
	Mean ± SD	71.4 ± 0.8
Carbamazepine (IS)	100	75.5 ±7.6

**Table 3:** Stability and dilution integrity data of LND in rabbit plasma

**Stability**	**Nominal conc. (ng/mL)**	**Measured conc. (ng/mL ± SD)**	**Precision (CV,%)**	**Accuracy (recovery,%)**
Bench top (6 h)	1.28	1.32 ± 0.08	6.13	103
	800	753 ± 26.24	3.50	94.1
Freeze thaw (3 cycle)	1.28	1.33 ± 0.05	3.77	104
	800	767 ± 23.76	3.10	95.8
In injector (48 h)	1.28	1.32 ± 0.06	4.24	103
	800	787 ± 38.06	4.80	98.3
30 days at 80°C	1.28	1.35 ± 0.04	2.79	105
	800	752 ± 16.24	2.16	94
Dilution integrity	360	373 ± 24.80	6.60	104
	720	681 ± 28.90	4.20	94.6

In dilution integrity study, the%accuracy of two and four times diluted sample was to 94.6 and 103.6%of the nominal concentration for LND. These results conclude that the dilution of the concentrated plasma sample upto four times maintains legibility and integrity of LND concentration.

#### Advantages of the proposed method over the reported methods

This study represents the first report describing the determination of LND in both animal and human plasma by UPLC-MS/MS method. The proposed method is superior to the previously reported LC-MS methods in terms of the sensitivity and simplicity as the method described herein is based on simple one step protein precipitation for sample preparation and isocratic flow of mobile phase containing acetonitrile:water:formic acid (65:35:0.1%, v/v/v) at a flow rate of 0.2 mL/min only. The run time was only 2.5 min which is suitable for high-throughput analysis.

## Conclusions

A novel simple, economical high-throughput and highly sensitive UPLC-MS/MS method was successfully developed and validated for the determination of LND in rabbit and human plasma. The method involved simple one step protein precipitation method for plasma sample preparation and short runtime (2.5 min) for analysis. The proposed method could be practical and reliable for pharmacokinetic and toxicokinetic study for LND in both animal and human.

## Abbreviations

LND: Lenalidomide;IS: Internal standard;UPLC: Ultra performance liquid chromatography;ESI: Electrospray ionization;MRM: Multiple reaction monitoring;FDA: Food and drug administration;EMEA: European Medicine Agency;LLOQ: Lower limit of quantification;LLOQ QC: Lower limit of quantification for quality control;LQC: Lower quality control;MQC: Middle quality control;HQC: High quality control

## Competing interests

The authors declare that they have no conflict of interests.

## Authors’ contributions

MI conducted the optimization and validation of the method, and prepared the draft manuscript. TAW participated in proposing the subject of the study, method development and validation, and discussion for the results. NYK contributed in reviewing the literature, method development, and revising the manuscript. IAD suggested the target analyte, contributed in literature review, results discussion, and revised the manuscript. All authors have read and approved the final manuscript.
